# Clinical decision support system for the management of osteoporosis compared to NOGG guidelines and an osteology specialist: a validation pilot study

**DOI:** 10.1186/s12911-019-0749-4

**Published:** 2019-02-01

**Authors:** Haukur T. Gudmundsson, Karen E. Hansen, Bjarni V. Halldorsson, Bjorn R. Ludviksson, Bjorn Gudbjornsson

**Affiliations:** 1 Department of Medicine, Landspitali - University Hospital, Reykjavik, Iceland; 2 Immunology and Centre for Rheumatology Research, Reykjavik, Iceland; 3 Landspitali - University Hospital, Reykjavik, Iceland; 4 Rheumatology Division, Department of Medicine, University of Wisconsin School of Medicine and Public Health, Madison, USA; 5 School of Science and Engineering, Reykjavik University, Reykjavik, Iceland; 6 The Faculty of Medicine, University of Iceland, Reykjavik, Iceland

**Keywords:** Clinical decision support system (CDSS), Clinical guidelines, Fracture risk, Osteoporosis, Treatment recommendations

## Abstract

**Background:**

Although osteoporosis is an easily diagnosed and treatable condition, many individuals remain untreated. Clinical decision support systems might increase appropriate treatment of osteoporosis. We designed the Osteoporosis Advisor (OPAD), a computerized tool to support physicians managing osteoporosis at the point-of-care. The present study compares the treatment recommendations provided by OPAD, an expert physician and the National Osteoporosis Guideline Group (NOGG).

**Methods:**

We performed a retrospective analysis of 259 patients attending the outpatient osteoporosis clinic at the University Hospital in Iceland. We entered each patient’s data into the OPAD and recorded the OPAD diagnostic comments, 10-year risk of major osteoporotic fracture and treatment options. We compared OPAD recommendations to those given by the osteoporosis specialist, and to those of the NOGG.

**Results:**

Risk estimates made by OPAD were highly correlated with those from FRAX (r = 0.99, 95% CI 0.99, 1.00 without femoral neck BMD; r = 0.98, 95% CI, 0.97, 0.99 with femoral neck BMD. Reassurance was recommended by the expert, NOGG and the OPAD in 68, 63 and 52% of cases, respectively. Likewise, intervention was recommended by the expert, NOGG, and the OPAD in 32, 37 and 48% of cases, respectively. The OPAD demonstrated moderate agreement with the physician (kappa 0.51, 95% CI 0.41, 0.61) and even higher agreement with NOGG (kappa 0.69, 95% CI 0.60, 0.77).

**Conclusion:**

Primary care physicians can use the OPAD to assess and treat patients’ skeletal health. Recommendations given by OPAD are consistent with expert opinion and existing guidelines.

## Background

Osteoporosis is a common disease that causes progressive deterioration of bone tissue resulting in lower bone density and an increased risk of an osteoporotic fracture [1].

Osteoporotic fractures carry a significant risk of mortality and morbidities, forming a growing burden in the ageing population [2].

Most osteoporotic fractures occur in the non-osteoporotic range (T-score > − 2.5) [3], emphasizing the influence of other risk factors on fracture incidence [4]. This has led to the development of several risk-prediction algorithms whose roles are to facilitate risk assessment [5]. Currently the best validated and the most widely used is the FRAX tool introduced in 2008 by the WHO Task Force [4]. The FRAX algorithm is based on data collected from several large international cohort studies on risk factors from Europe, North America, Asia and Australia, and has been validated in many independent cohorts [6].

Risk-assessment tools help identify individuals who need treatment [6], but their usefulness depends on the clinician’s ability to correctly translate their results into clinical practice congruent with evidence-based guidelines [7, 8]. Osteoporosis is a highly treatable condition but despite clinical guidelines being widely available [9], many individuals that are at substantial risk of fracture still go untreated [10, 11]. Promoting management guidelines is essential, but that alone is unlikely to solve this problem, the fact being that only 11% of patients suffering from a fragility fracture of the hip are started on bone protective treatment as a secondary prevention [12]. Additionally, non-adherence to treatment is frequently reported [13]. Consequently, there is obviously room for improvement in the management of osteoporosis, especially at the primary care level [14].

Closing this treatment gap is a priority that involves improving selection of patients at risk of fracture for DXA scanning, securing optimal treatment when indicated and providing follow-up that encourages patient adherence to treatment. Given limited resources allocated to health care it is equally important to avoid performing unnecessary and costly DXA scans among people with good bone health. Our objective was to close the gap in osteoporosis care, by identifying individuals at high fracture risk to begin appropriate treatment, and identifying individuals at low risk of fracture who would not benefit from DXA scanning. We thus developed the Osteoporosis Advisor or OPAD (http://www.expeda.is), a computerized clinical decision support system to assess and treat osteoporosis in a primary care setting [15]. The OPAD was developed as a collaboration between a commercial start-up company (Expeda), The University of Iceland and Reykjavik University. Although the source code of the OPAD is currently proprietary, the OPAD system is openly accessible to all practicing doctors in Iceland. OPAD provides a patient’s 10-year fracture probability (with or without BMD values) using a speedometer-like output that users can easily understand. The OPAD also notes whether a patient would benefit from BMD measurement by DXA. OPAD provides patients with lifestyle and treatment recommendations to reduce their fracture risk, incorporating country-specific guidelines for therapy. Finally, OPAD provides guidance on whether to refer to an osteoporosis specialist.

We have previously described the technical design of the OPAD [15].

The objective of this study was to compare recommendations provided by OPAD both to a osteoporosis specialist and the current management guidelines from the NOGG (National Osteoporosis Guideline Group) [16]. To accomplish this objective, we retrospectively reviewed the DXA reports and medical records of patients undergoing routine DXA at one university setting in 2012.

## Methods

We performed a retrospective cohort study, reviewing the DXA reports and medical records of 308 consecutive patients undergoing BMD measurement in 2012 at the University Hospital of Iceland Osteoporosis Clinic. We excluded individuals < 40 and > 90 years old (*n* = 13) and those receiving osteoporosis therapies (*n* = 36), as FRAX was not developed to estimate fracture risk in these individuals.

In the remaining 259 patients, we gathered data from two major sources:

1. *A standardized questionnaire,* which all patients completed prior to DXA scanning. The questionnaire assessed age, gender, menopausal status, past DXA scans, hormone replacement therapy, medication usage (including corticosteroids), current smoking, family history of osteoporosis, rheumatoid arthritis, secondary causes of osteoporosis, previous fracture, calcium and vitamin D intake and exercise.
2. *Detailed DXA reports*, stating the height and weight as measured at the time of BMD testing, BMD values from the hip and lumbar spine, and written comments made in a guideline-driven fashion by a physician with expertise in osteoporosis management. Reports contained suggestions for treatment, suitable follow-up, lifestyle modifications and additional diagnostic tests, if indicated.

All patients had BMD measured with the same Hologic QDR 4500A DXA scanner. BMD measurements at the lumbar spine (L2-L4) and the femoral neck were performed. BMD was classified according to WHO definitions (osteopenia, T-score − 1.1 to − 2.4 and osteoporosis, T-scores ≤ − 2.5). Patients taking glucocorticoid therapy (prednisolone > 7.5 mg/day) were diagnosed with glucocorticoid induced osteoporosis if their T-score was below − 1.5.

Using measured BMD and information gathered from the questionnaire, we calculated each person’s 10-year risk of major osteoporotic fracture with FRAX and OPAD. We accessed the online tool on the FRAX website (http://www.shef.ac.uk/FRAX/), to determine 10-year fracture risk estimates with, and without, femoral neck BMD. In a similar fashion, we determined fracture risks using the OPAD with and without femoral neck BMD. As OPAD takes into account several protective factors in its risk prediction (estrogen or testosterone therapy, vitamin D, calcium and exercise), risk estimates were performed while omitting these protective factors, thus, using only the risk factors shared by FRAX and OPAD.

Each case was entered into the OPAD, recommendations were recorded and then grouped into two categories: “reassurance” and “intervention indicated”. Likewise, FRAX scores for each case were evaluated according to the age variable interventional thresholds proposed by the National Osteoporosis Guideline Group (NOGG) [16], and grouped into the two categories (reassurance and intervention). Finally, the expert physician’s recommendations in each report were grouped into the two categories of reassurance and therapy.

### Statistical methods

We performed statistical analyses using SPSS software version 22 (Armonk, NY: IBM Corp). We summarized descriptive data using the mean and standard deviation. We compared estimates of fracture risk by FRAX and OPAD using paired t-tests, Pearson correlation coefficients. We used the Bland-Altman test to assess numeric agreement within and between the two fracture risk tools. We assessed inter-rater agreement (reassurance versus treatment for a given individual) between the OPAD, FRAX and expert physician using the Kappa test statistic.

## Results

Of the total study population (*n* = 259), 30 were male and 229 were female, including 182 post-menopausal women. Cases’ mean age was 62 ± 10 years old. One hundred twenty cases had previously undergone BMD measurement. The majority of cases reported sufficient intake of calcium, vitamin D and adequate exercise (Table 1). Sixty-eight individuals (26%) suffered a prior fragility fracture and 64 individuals (25%) reported a family history of osteoporosis. Twenty-five cases (10%) had secondary osteoporosis. We summarize further characteristics of the study population in Table 1.

**Table 1:** Demographic variables of the cohort. Values are presented as means ± SD or numbers (%)

Characteristic	Women	Men	Total sample
Population	229 (88%)	30 (12%)	259
Age (years): mean ± SD	61.6 ± 10.2	66,1 ± 11.1	62 ± 10.35
BMI (kg/m^2^): mean ± SD	25.6 ± 4.6	24.7 ± 2.7	25.5 ± 4.43
Prior scan: n (%)	120 (52%)	10 (33%)	130 (50%)
Menopausal status: n (%)			
Pre-menopausal	25 (11%)		
Peri-menopausal	22 (10%)		
Post-menopausal	182 (80%)		
Receiving hormonal replacement	36 (16%)		
Current smoking: n (%)	26 (11%)	5 (17%)	31 (12%)
Glucocorticoids: n (%)	27 (12%)	7 (23%)	34 (13%)
Family history: n (%)	59 (26%)	5 (16%)	64 (25%)
Parent hip-fracture: n (%)	2 (1%)	0 (0%)	2 (1%)
Previous fracture: n (%)	59 (26%)	9 (30%)	68 (26%)
Rheumatoid arthritis: n (%)	19 (8%)	0 (0%)	19 (7%)
Secondary osteoporosis: n (%)	25 (11%)	0 (0%)	25 (10%)
Alcohol users: n (%)	0 (0%)	2 (7%)	2 (1%)
Sufficient calcium intake: n (%)	164 (72%)	23 (77%)	187 (72%)
Sufficient vitamin-D intake: n (%)	174 (76%)	23 (77%)	197 (76%)
Regular exercise: n (%)	114 (50%)	18 (60%)	132 (51%)

One hundred eighty individuals (70%) had osteopenia and 52 (20%) had osteoporosis based on the BMD values measured at either the femoral neck or lumbar spine. T-scores were higher at the lumbar spine, compared to the femoral neck (− 1.1 vs. − 1.3, *p* = 0.044). We summarize BMD data in Table 2.

**Table 2:** Bone mineral density and osteoporosis (T-value ≤ −2.5) or osteopenia (T-value −1 - -2.5) according to WHO definitions

DXA site	Women (*n* = 229)	Men (*n* = 30)	Total (*n* = 259)
Femoral neck			
Osteoporosis: n (%)	25 (11%)	2 (7%)	27 (10%)
Osteopenia: n (%)	120 (52%)	18 (60%)	138 (53%)
T-score mean ± SD	−1.3 ± 1.0	−1.3 ± 0.7	-1.3 ± 0.9
Lumbar spine			
Osteoporosis: n (%)	31 (14%)	6 (20%)	37 (14%)
Osteopenia: n (%)	92 (40%)	17 (57%)	109 (42%)
T score mean ± SD	−1.1 ± 1.5	− 1.4 ± 1.6	−1.1 ± 1.5
Neck or spine			
Osteoporosis: n (%)	45 (20%)	7 (23%)	52 (20%)
Osteopenia: n (%)	155 (68%)	25 (83%)	180 (70%)

### Fracture risk

**Fig. 1:**
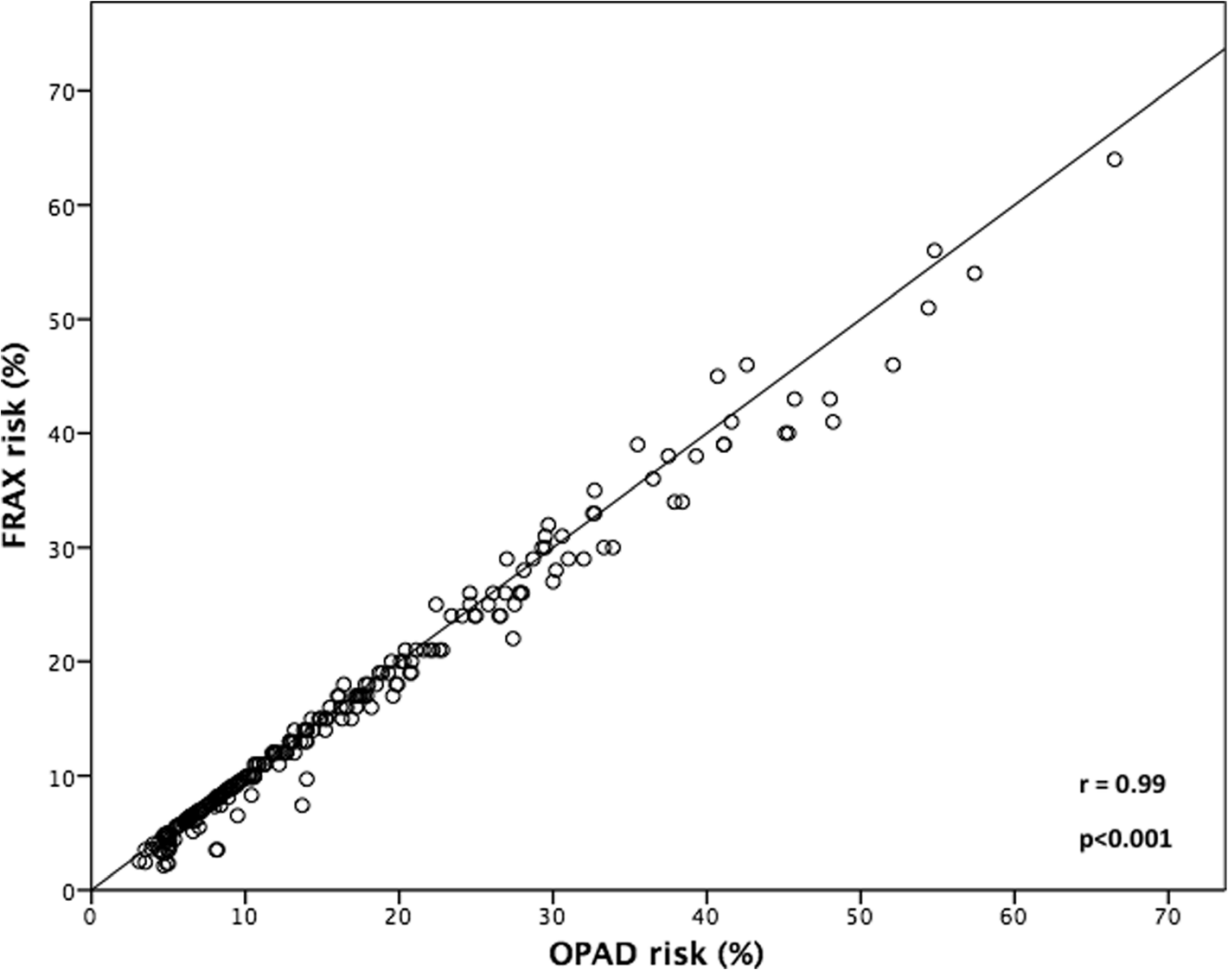
Ten-year risk estimates (without BMD values) from OPAD (x-axis) compared to FRAX (y-axis)

Using FRAX, the mean 10-year risk of major osteoporotic fracture for the cohort was 15.4 ± 11.3% relying solely on risk factors and 13.6 ± 9.4% when femoral neck BMD was included. Using OPAD, fracture risk was similar (16 ± 11.6% without, and 15.4 ± 11.0% with femoral neck BMD) (Table 3). Risk estimates made by OPAD were highly correlated with those from FRAX (r = 0.99, 95% CI 0.99, 1.00 without femoral neck BMD; r = 0.98, 95% CI, 0.97, 0.99 with femoral neck BMD, Fig. 1).

**Table 3:** Estimates of the 10-year risk (%) of major osteoporotic fracture as calculated by FRAX and OPAD

Risk estimation tool	Women (*n* = 229)	Men (*n* = 30)	Total (*n* = 259)
FRAX mean (SD)			
With BMD (%)	14 ± 10%	11 ± 5%	14 ± 9%
Without BMD (%)	16 ± 12%	11 ± 5%	15 ± 11%
OPAD mean (SD)			
With BMD (%)	16 ± 12%	12 ± 5%	15 ± 11%
Without BMD (%)	17 ± 12%	12 ± 6%	16 ± 12%

**Fig. 2:**
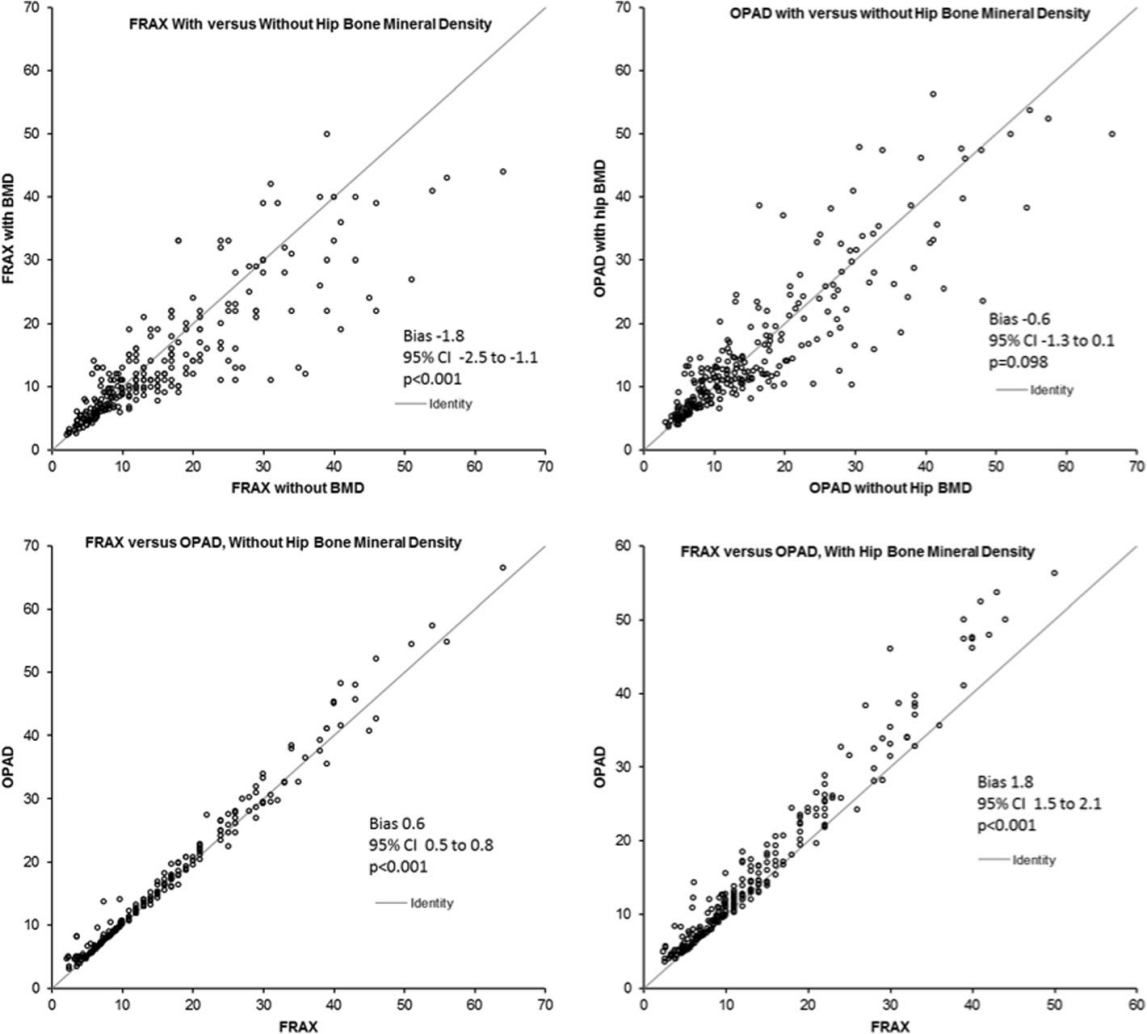
Bland-Altman plots comparing 10-year fracture risk estimates of OPAD and FRAX with and without BMD values

Bland-Altman analysis revealed that the estimated probability of fracture using FRAX with BMD was lower by 1.8% (95% CI, − 2.5, − 1.1%, *p* < 0.001), compared to the estimated fracture risk using FRAX without hip BMD values (Fig. 2). OPAD estimates of fracture risk were similar, whether determined with or without hip BMD (*p* = 0.098). Without BMD values, OPAD overestimated fracture risk compared to FRAX (bias 0.63, 95% CI 0.5, 0.8%, *p* < 0.001). Likewise, when using hip BMD values to estimate fracture risk, the OPAD overestimated risk compared to FRAX (bias 1.8, 95% CI -1.5%, 2.1, *p* < 0.001). However, while the OPAD fracture risk estimates were significantly higher than FRAX based on statistical tests, the numeric differences in fracture risk using the two tools were very small.

**Fig. 3:**
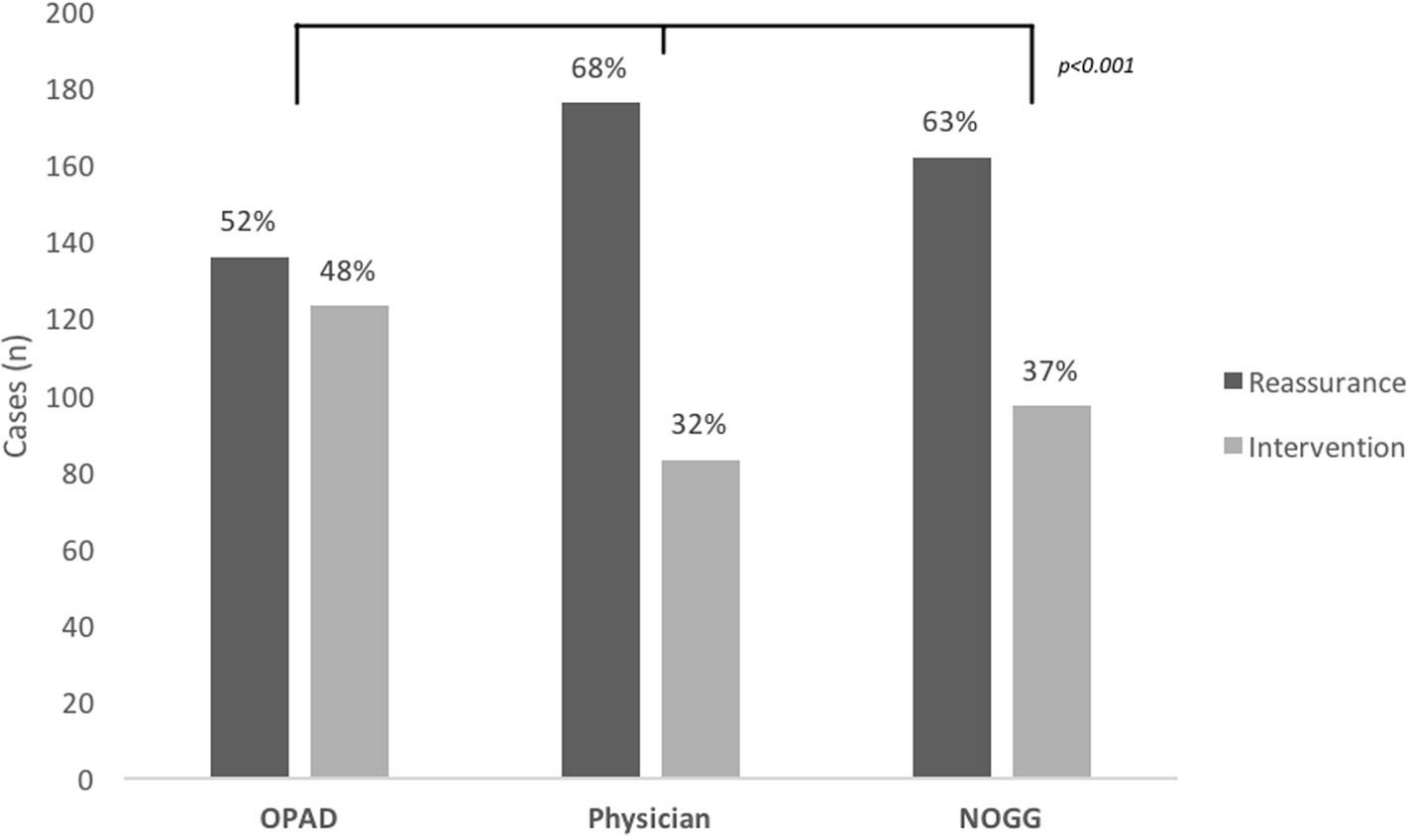
Frequency of recommendations made by OPAD, physician and NOGG

**Fig. 4:**
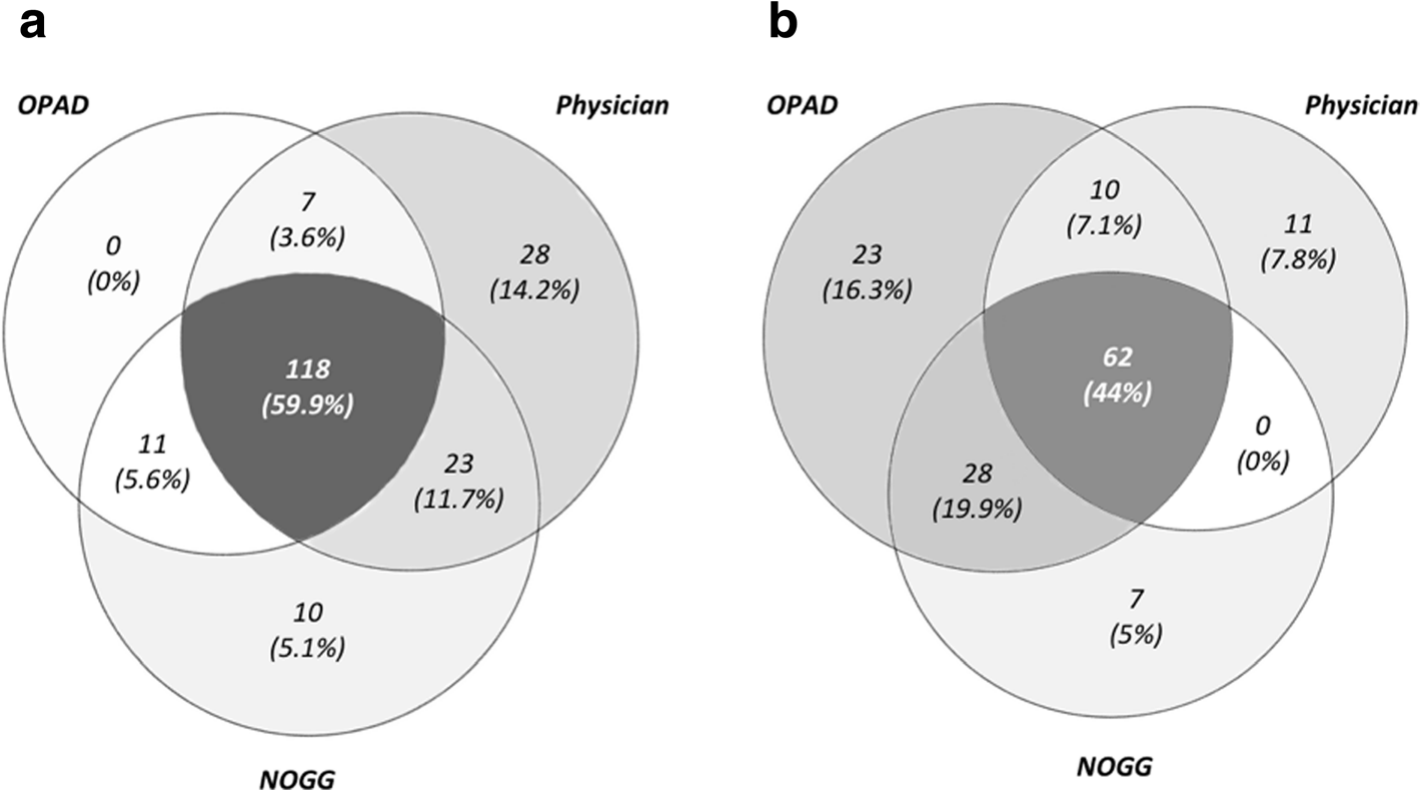
Agreement of intervention decisions made by OPAD to physician and NOGG guidelines. Venn diagram showing agreement in the decision of reassurance (**a**) vs the decision of intervention (**b**)

The physician, NOGG and OPAD recommended reassurance in 68, 63 and 52% of 259 cases, respectively. Conversely, the physician, NOGG and OPAD recommended intervention in 32, 37 and 48% of cases, respectively (Fig. 3). We used kappa test statistics to assess within-subject agreement to reassure or treat, using the OPAD or FRAX with NOGG directed therapy. The OPAD demonstrated moderate agreement with the physician (kappa 0.51, 95% CI 0.41, 0.61) and even higher agreement with NOGG (kappa 0.69, 95% CI 0.60, 0.77). The physician was in moderate agreement with NOGG (kappa 0.53 (95% CL 0.415, 0.629) (Table 4 and Fig. 4).

**Table 4:** Inter-rater agreement using percentage and kappa statistics

OPAD	Physician	NOGG
Agreement (%)	76%	85%
Kappa (95% CI)	0.51 (0.41–0.61)	0.69 (0.60–0.77)

## Discussion

Osteoporosis is a common disease that remains asymptomatic until a fragility fracture occurs; representing a silent epidemic disorder. The only way to diagnose osteoporosis prior to fracture is by measuring BMD [17]. It is of importance to identify those who are at high risk of fragility fractures and thus could benefit from DXA evaluation and treatment intervention [18]. Several interest organizations including the National Osteoporosis Foundation (NOF) recommend BMD screening for women starting at the age of 65 and for men starting at age 70 [19]. Despite good access to DXA, effective treatments and widely published treatment guidelines, patients remain untreated, even after suffering hip fractures [10]. Therefore, we developed a user-friendly clinical tool to support primary care providers in decisions to screen for and treat osteoporosis. We compared the performance of the OPAD to that of FRAX, the most widely used and best-validated of several fracture risk calculators [20]. In our study, the risk evaluations made by OPAD were highly correlated with calculations made by FRAX. Correlation between FRAX and OPAD remained high even after additional protective factors (hormonal therapy, vitamin D, calcium intake and exercise) where included in the risk calculations made by OPAD (data not shown).

Although low BMD is strongly associated with increased fracture risk, it is essential to incorporate other risk factors to sufficiently evaluate and select at-risk patients for bone protective treatment [21]. The FRAX risk assessment tool is intended to aid the clinician in the decision to initiate preventive treatment by providing the patient’s ten-year risk of an osteoporotic fracture. However, there are several limitations of FRAX that have already been discussed [22, 23]. Furthermore, the fracture risk given by FRAX still needs to be interpreted in accordance with the terms in the current management guidelines. Although those guidelines are readily available, too many patients-at-risk are receiving sub-optimal treatment [24]. Consequently, there is obviously room for improvement in the management of osteoporosis, especially at the primary care level [14].

The agreement of the recommendations given by the OPAD to those given by the osteoporosis specialist and NOGG guidelines are of importance. Our study shows that recommendations made by OPAD are in good agreement to specialist advice and even stronger agreement to the NOGG intervention thresholds. With zero patients being recommended “reassurance” in discordance to either a specialist or NOGG, our study shows the OPAD system does not overlook individuals who need bone protective treatment. When OPAD recommended active treatment intervention, the majority of cases were in agreement to both the specialist and NOGG but 16% (*n* = 23) were discordant, demonstrating a slightly more aggressive stance for treatment. In the most of those cases (*n* = 23, 74%), the OPAD system had made the recommendation to seek expert advice. To facilitate comparison those recommendations where classified as an intervention, thus accounting for some cases of discrepancy between OPAD and the expert physician. The rest of the cases (*n* = 6) are explained by OPAD’s slight overestimation in its risk estimations compared to FRAX as revealed by Bland-Altman’s analysis. Several other reasons might explain why OPAD and the clinician disagreed, e.g. additional clinical information that the specialist had access to but was not included in OPAD.

More important is the fact that OPAD not only gives the fracture risk, but also recommend clinical decisions based on guidelines [15]. Thus, health care professionals do not need to know the guidelines in order to use OPAD. The software design of OPAD allows it to be effortlessly integrated in to different EMR (Electronic Medical Record) systems so to fit easily in to existing workflow of health care providers, further enabling it’s use at the point of care. Furthermore, with a systematic approach using a CDSS (Clinical Decision Support System) such as OPAD in the osteoporosis care, we might reduce the cost and improve the utilization of diagnostic resources. Further health economic studies on this issue are of interest.

It is important to note that the OPAD system is not solely a risk calculator like FRAX, but a fully featured CDSS that evaluates patient risk factors and BMD values to provide the clinician with treatment recommendation, and advice on proper follow-up. OPAD also highlights primary prevention and selects individuals for further work-up, such as BMD measurement or referral to an osteoporosis specialist [15]. In the present study, the OPAD tool was tested on an Icelandic population, but using different epidemiological data, OPAD is able to run in up to ten different national specific datasets [15].

Our study has both strengths and limitations. The strength of this study is that it is based on a real-life data, gathered from a BMD centre that does not require a doctor’s referral for post and peri-menopausal females and should therefore be a better representation of a typical primary care population compared to a strict tertiary referral centre. Furthermore, the osteoporosis specialist who analysed the DXAs was unaware of the present study, minimizing potential interpretation or performance bias. In addition, the risk evaluation and recommendations given by the OPAD are concordant with those from FRAX and an osteology specialist. Thus, the data set and results are of interest for general practice. The limitations of this study are its retrospective design, small sample size, non-random sampling of a racially homogenous study population and that it was conducted at a single academic centre.

The OPAD is under active development and adapts to ever changing management guidelines. Further patient factors may be incorporated into the risk assessment model of OPAD, such as loss of height, history of falls and it is even possible to add individual genotypes to improve the fracture risk estimation. We believe the positive observations made from this limited retrospective study warrant the undertaking of a larger multi-centred prospective validation study in a primary care setting.

## Conclusion

We conclude that fracture risk estimations made by the OPAD system are equivalent to risk calculations made by FRAX. Treatment recommendations by OPAD are accurate and consistent with NOGG guidelines and those given by a practicing specialist. With the use of a clinical decision aid such as OPAD, we offer a new approach to reduce gaps in screening and treatment for older patients, many of whom suffer from osteoporosis. Our tool is novel as it provides an output that is user friendly and easily understood by both patients and primary care providers, regardless of their knowledge of treatment guidelines.

## Abbreviations

BMD: Bone mineral density
CDSS: Clinical decision support system
DXA: Dual-energy X-ray absorptiometry
EMR: Electronic medical record
NOGG: National Osteoporosis Guideline Group
OPAD: Osteoporosis advisor
WHO: World Health Organization

## References

[1] Riggs BL, Melton LJ (1995). The worldwide problem of osteoporosis: insights afforded by epidemiology. Bone.

[2] Ström O, Borgström F, Kanis JA, Compston J, Cooper C, McCloskey EV (2011). Osteoporosis: burden, health care provision and opportunities in the EU. Arch Osteoporos Springer-Verlag.

[3] Siris ES, Chen Y-T, Abbott TA, Barrett-Connor E, Miller PD, Wehren LE (2004). Bone mineral density thresholds for pharmacological intervention to prevent fractures. Arch Intern Med.

[4] Kanis JA, Johnell O, Oden A, Johansson H, McCloskey E (2008). FRAX™ and the assessment of fracture probability in men and women from the UK. Osteoporos Int.

[5] Baim S, Leslie WD (2012). Assessment of fracture risk. Curr Osteoporos Rep.

[6] MacLean FR, Thomson SA, Gallacher SJ (2012). Using WHO-FRAX to describe fracture risk: experience in primary care. Scott Med J.

[7] Kanis JA, Hans D, Cooper C, Baim S, Bilezikian JP, Task Force of the FRAX Initiative (2011). Interpretation and use of FRAX in clinical practice. Osteoporos Int.

[8] Lewiecki EM (2009). Managing osteoporosis: challenges and strategies. Cleve Clin J Med.

[9] Elvey MH, Pugh H, Schaller G, Dhotar G, Patel B, Oddy MJ (2014). Failure in the application of fragility fracture prevention guidelines. Ann R Coll Surg Engl.

[10] Nguyen TV, Center JR, Eisman JA (2004). Osteoporosis: underrated, underdiagnosed and undertreated. Med J Aust.

[11] Vestergaard P, Rejnmark L, Mosekilde L (2005). Osteoporosis is markedly underdiagnosed: a nationwide study from Denmark. Osteoporos Int.

[12] Baba T, Hagino H, Nonomiya H, Ikuta T, Shoda E, Mogami A (2015). Inadequate management for secondary fracture prevention in patients with distal radius fracture by trauma surgeons. Osteoporos Int.

[13] Rabenda V, Mertens R, Fabri V, Vanoverloop J, Sumkay F, Vannecke C (2008). Adherence to bisphosphonates therapy and hip fracture risk in osteoporotic women. Osteoporos Int.

[14] Taylor JC, Sterkel B, Utley M, Shipley M, Newman S, Horton M (2001). Opinions and experiences in general practice on osteoporosis prevention, diagnosis and management. Osteoporos Int.

[15] Halldorsson BV, Bjornsson AH, Gudmundsson HT, Birgisson EO, Ludviksson BR, Gudbjornsson B (2015). A clinical decision support system for the diagnosis, fracture risks and treatment of osteoporosis. Comput Math Methods Med.

[16] Compston J, Bowring C, Cooper A, Cooper C, Davies C, Francis R, et al. Diagnosis and management of osteoporosis in postmenopausal women and older men in the UK: National Osteoporosis Guideline Group (NOGG) update 2013. Maturitas. 2013;75:392–6. 10.1016/j.maturitas.2013.05.013 23810490

[17] Rachner TD, Khosla S, Hofbauer LC (2011). Osteoporosis: now and the future. Lancet.

[18] Silverman SL (2007). Selecting patients for osteoporosis therapy. Ann N Y Acad Sci.

[19] Cosman F, de Beur SJ, LeBoff MS, Lewiecki EM, Tanner B, Randall S (2014). Clinician’s guide to prevention and treatment of osteoporosis. Osteoporos Int.

[20] McCloskey E, Johansson H, Oden A, Kanis JA (2012). Fracture risk assessment. Clin Biochem.

[21] Kanis JA, McCloskey EV, Johansson H, Cooper C, Rizzoli R, Reginster JY (2013). European guidance for the diagnosis and management of osteoporosis in postmenopausal women. Osteoporos Int.

[22] Lewiecki EM, Compston JE, Miller PD, Adachi JD, Adams JE, Leslie WD, et al. FRAX(®) Bone Mineral Density Task Force of the 2010 Joint International Society for Clinical Densitometry & International Osteoporosis Foundation Position Development Conference. 2011. pp. 223–225. 10.1016/j.jocd.2011.05.018 21810529

[23] Silverman SL, Calderon AD (2010). The utility and limitations of FRAX: a US perspective. Curr Osteoporos Rep.

[24] Naranjo A, Rosas J, Ojeda S, Salas E, CANAL group (2013). Management of osteoporosis in primary care before and after the result of densitometry: treatments in real practice versus the recommended by guidelines. CANAL study. Reumatol Clin.

